# Disease-specific signatures of circulating extracellular vesicles detected by the surface plasmon resonance imaging: a pilot study

**DOI:** 10.20517/evcna.2024.82

**Published:** 2025-02-11

**Authors:** Tatsuki Shibuta, Yukichi Takada, Shiori Nishinosono, Seiko Yasuda, Yasuhiro Ono, Yoshitaka Hirooka, Daisuke Irikura, Kensuke Saito, Tsukuru Umemura

**Affiliations:** ^1^Department of Medical Technology and Sciences, International University of Health and Welfare, Fukuoka 831-8501, Japan.; ^2^Department of Diabetes and Metabolism, Kouhoukai Takagi Hospital, Fukuoka 831-0016, Japan.; ^3^Hypertension and Heart Failure Center, Kouhoukai Takagi Hospital, Fukuoka 831-0016, Japan.; ^4^Graduate School, International University of Health and Welfare, Fukuoka 831-8501, Japan.; ^5^HORIBA, Ltd, Kyoto 601-8510, Japan.

**Keywords:** Extracellular vesicles, surface plasmon resonance imaging, diabetes, hypertension, blood cell

## Abstract

**Aim:** Cells in the human body release extracellular vesicles (EVs) into fluids, such as plasma, urine, and cerebrospinal fluid. EVs express tetraspanin family proteins (e.g., CD63, CD9, and CD81) and cell-specific antigens on their surface as common and specific markers, respectively. In this study, we hypothesized that the profile of blood cell-derived circulating EVs could reveal both common and specific pathophysiology in atherogenic diseases.

**Methods:** Using surface plasmon resonance imaging (SPRi), we analyzed EVs surface molecules and identified circulating EVs in healthy controls (*n* = 18), patients with type 2 diabetes mellitus (T2DM; *n* = 71), and those with hypertension (HT; *n* = 47).

**Results:** Patients with T2DM and HT exhibited distinct EV profiles: (i) CD9, CD110, CD20, activin receptor type-2A (AcvRIIA), Duffy antigen receptor for chemokine, and CD44 positive EVs were upregulated in T2DM; (ii) CD9, Maackia amurensis agglutinin lectin binding molecules (MBM), CD20, AcvRIIA, and CD44 positive EVs were upregulated in HT. By analyzing an appropriate set of three antigens or using dimensional reduction clustering, we were able to clearly differentiate between T2DM, HT, and control groups. In some patients, disease severity correlated with CD44 and CD20 in T2DM and MBM and AcvRIIA in HT.

**Conclusion:** Our findings demonstrate that profiling of circulating EVs via the SPRi method offers a novel approach for diagnosing and monitoring human diseases.

## INTRODUCTION

Extracellular vesicles (EVs) are nanoparticles, 50-200 nm in size, released by nearly all cell lineages and found in extracellular fluids, including plasma, urine, and cerebrospinal fluid^[1-3]^. EVs carry proteins, ribosomal RNA, messenger RNA (mRNA), and micro RNA (miRNA), transporting these materials to recipient cells. This makes EVs vital for cell-cell communication and maintaining homeostasis^[4]^. EVs from different parent cells have distinct contents and functions; for instance, EVs from immune cells convey antigen information^[5]^, whereas those from tumor cells play a role in metastasis and angiogenesis^[1,2,4]^. Thus, identifying the origins of circulating EVs is crucial for understanding their biological and pathophysiological functions. The identification of parent cells has been achieved through various methods, such as flow cytometry to analyze surface molecules, protein analysis via western blot or proteomics^[6]^, and transcriptome analysis^[7]^. To obtain disease-specific profiles of circulating EVs, we used surface plasmon resonance imaging (SPRi) to analyze their surface antigens and sugar chains. SPRi measures changes in reflected light intensity due to intermolecular binding on the biochip surface (such as ligand-antibody-lectin, single-stranded DNAs, or drug-protein interactions)^[8-12]^. On a gold-coated surface, surface plasmons (collective oscillations of conduction electrons) interact with light to create a surface plasmon wave. Under total internal reflection, these surface plasmons couple with the evanescent wave, causing resonance. The binding of molecules to ligands on the gold-coated surface affects this resonance, leading to changes in the refractive index, and SPRi detects these changes in real time. As ligands attached to spotted antibodies can be removed by washing with a regeneration buffer, SPRi allows for repeated analysis of multiple samples, up to a hundred, reducing operational costs per sample^[13]^. Recently, Gorodkiewicz’s group has reported on the measurement of IL-6^[14]^, poly(ADP-ribose) polymerase 1 (PARP-1) protein^[15]^, and cathepsin S^[16]^ in plasma using SPRi, showing the increasing application of SPRi in the medical field. EVs display ubiquitous (e.g., CD63, CD9, CD81) and specific markers from their parent cells on their surface membranes^[2,3]^. Therefore, analyzing the combined surface antigens on EVs offers a promising method to detect disease-specific changes. Several researchers have established the use of quantitative analysis of EVs with the SPRi method^[17,18]^, demonstrating superior quantitative performance compared to ELISA^[19]^. Lopez Baltazar *et al.* conducted a detailed study on the detectability of EVs using surface plasmon resonance technique, focusing on the tetraspanin family of EV membrane proteins^[20]^. Picciolini *et al.* analyzed CD171, PLP1, and CD11b on the membrane surface of EVs in the plasma of patients with Alzheimer’s Disease using surface plasmon resonance technique and demonstrated that these markers are potential new biomarkers^[21]^. Other optical methods for detecting EVs are also advancing, including nano-flow cytometry, which is now commercially available and holds promise for the future, although it requires careful attention to labeling and nonspecific reactions, as verified by Brealey *et al.*^[22]^*.* A new optical EV detection method using surface-enhanced Raman scattering (SERS) is being developed^[23]^. Although the SPRi method has been used to analyze EVs from the serum of patients with tumors^[18,24]^, there are no studies applying this technique to lifestyle-related diseases, such as diabetes, hypertension (HT), and cardiovascular disease, which are on the rise globally.

Li *et al.* estimated that 99.8% of circulating EVs originate from hematopoietic cells^[25]^. If blood cell-derived EVs exhibit disease-specific profiles, analyzing circulating EVs could aid in diagnosis. As the global elderly population increases, addressing chronic diseases, such as lifestyle-related conditions and various cancers, becomes increasingly critical. In particular, type 2 diabetes mellitus (T2DM) and HT are common, high-risk conditions for atherosclerosis and vascular endothelial damage, with prevalence rates of 6.1% in 2021^[26]^ for T2DM and 49%-59% of individuals aged 30-79 years in 2019 for HT^[27]^. Both conditions contribute to atherosclerosis, which can lead to cardiovascular events, stroke, and chronic kidney disease^[28]^. EV-related disruptions in homeostasis play a role in promoting atherosclerosis by causing endothelial dysfunction, vascular calcification, and the formation of unstable plaques or thrombi^[29]^. Current methods for detecting atherosclerosis and vascular endothelial damage include physiological tests, such as ankle-brachial index, cardio-ankle vascular index, and flow-mediated dilatation, but no effective blood biomarkers have yet been identified. We hypothesized that the profile of circulating blood cell-derived EVs reflects both common and specific aspects of atherogenic disease pathophysiology. In this study, we used SPRi technology to analyze the profiles of circulating EVs in patients with T2DM and HT.

## METHODS

### EV purification from serum samples for SPRi

**Figure 1:**
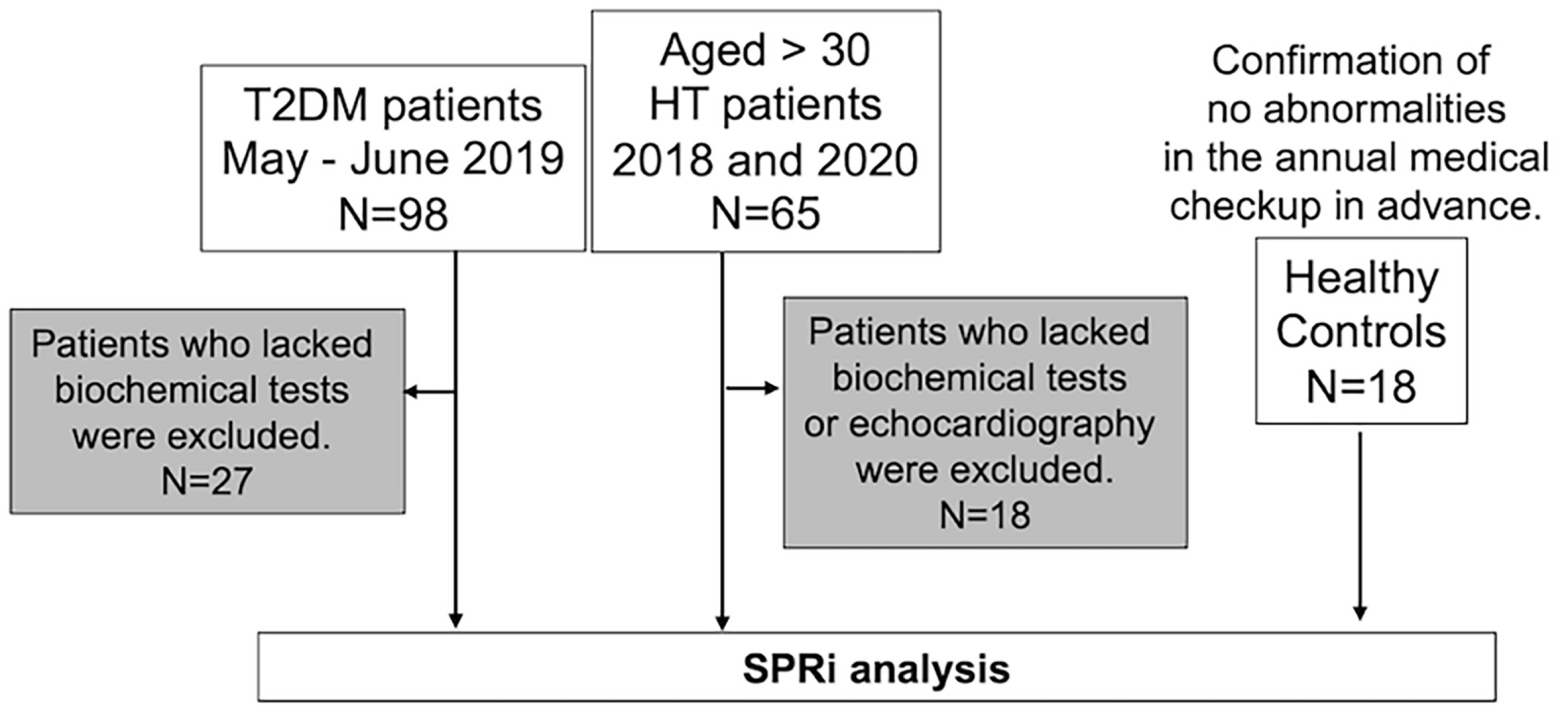
Flowchart of the patient selection process. T2DM: Type II diabetes mellitus; HT: hypertension.

Serum samples were collected from healthy controls (*n* = 18; 10 males; aged 23-70 years, median age of 41 years, normal vital signs, and normal laboratory results) and from patients with T2DM (*n* = 71) and HT (*n* = 47) at Takagi Hospital and International University of Health and Welfare [Table 1]. The inclusion criteria: all patients diagnosed with T2DM by Japanese Clinical Practice Guideline for Diabetes 2019 [plasma glucose levels: fasting ≥ 126 mg/dL, 2-h 75 g oral glucose tolerance tests ≥ 200 mg/dL or casual ≥ 200 mg/dL, and HbA1c ≥ 6.5%]^[30]^, and patients aged > 30 years diagnosed with HT by the Japanese Society of Hypertension (JSH) revised Guidelines for the Management of Hypertension 2014 and 2019 [office systolic blood pressure ≥ 140 mmHg and/or diastolic blood pressure ≥ 90 mmHg]^[31,32]^. The study included 98 patients with T2DM who visited the Department of Diabetes and Metabolism at Takagi Hospital between May and June 2019 and 65 patients with HT who visited the Hypertension and Heart Failure Center at Takagi Hospital between January 2018 and 2020. However, 27 patients with T2DM and 18 patients with HT were excluded due to unavailability of required laboratory tests [Figure 1]. Informed consent was obtained orally in accordance with the Declaration of Helsinki, and the study was approved by the Kouhoukai Ethics Committee (No. 224 and 333) and the Ethics Committee of the International University of Health and Welfare (18-Ifh-066). All participants were recruited specifically for this study. The median duration of therapy for patients with T2DM was 16 years (ranging from 2 to 69 years). Among the patients with T2DM, 38 were using dipeptidyl peptidase-4 inhibitors and 10 were using glucagon-like peptide-1 receptor agonists. By the attending physician, diabetic retinopathy was assessed through fundoscopy and diabetic nephropathy was diagnosed with urine albumin > 30 mg/gCr as an indicator^[33]^. Patients with HT did not receive any medication, and we classified them into non-risk [refined risk score (RS) < 10; *n* = 10], low- (RS = 21-30; *n* = 15), moderate- (RS = 31-40; *n* = 13), and high-(RS > 40; *n* = 9) groups for cardiovascular disease based on a RS method from the Japan Arteriosclerosis Longitudinal Study^[34]^. Serum samples were filtered through a 0.22-µm filter (Minisart RC, Sartorius, Goettingen, Germany) and stored at -80 °C until use. To isolate EVs, 25 µL of ExoQuick® (System Biosciences, CA, USA) was added to 100 µL of serum and incubated at 4 °C for 30 min. Following centrifugation at 1,500 × *g* at 4 °C for 30 min, the supernatant was discarded, and the EVs pellet was resuspended in 100 µL of 0.1% casein (C6554, Sigma-Aldrich, MO, USA) in Phosphate-buffered saline (PBS; 9.57 mM, pH 7.35, without magnesium and calcium). The sample for SPRi analysis was prepared by diluting this EV solution to 40-fold.

**Table 1:** Patients’ characteristics and laboratory data

	**Parameters**	**Median (Range)**
Healthy controls^*^	N	18
	Age (years)	41 (23-70)
	Gender (% male)	55.6
Type II diabetes mellitus	N	71
	Age (years)	68 (32-93)
	Gender (% male)	64.7
	Hemoglobin A1c (%)	7.6 (5.3-11.0)
	Glycoalbumin (%)	20.4 (12.5-36.5)
	eGFR (mL/min/1.73m^2^)	71 (7-166)
	Serum creatinine (mg/dL)	0.78 (0.34-7.08)
	Urine albumin (mg/gCr)	28.7 (2.3-2,390)
Hypertension	N	47
	Age (years)	58 (38-79)
	Gender (% male)	65.9
	Systolic blood pressure (mmHg)	163.5 (130-207)
	Diastolic blood pressure (mmHg)	99.2 (67-134)
	Brain natriuretic peptide (pg/mL)	15.3 (5.8-90.6)
	Noradrenalin (pg/mL)	322 (142-647)

^*^Healthy controls had an annual medical checkup (complete blood counts, biochemical tests, urinalysis, electrocardiogram, and blood pressure) to ensure no abnormalities before the study. eGFR: Estimated glomerular filtration rate; N: number.

### EV measurement by SPRi

EVs were detected using OpenPlex (Label-Free Biomolecular Interaction Analyzer, Horiba Scientific). For each experiment, running buffer (0.1% casein in PBS) and regeneration buffer (4 M MgCl_2_; Nacalai Tesque, Kyoto, Japan) were used. A 300 µL sample was injected into the instrument at a flow rate of 25 µL/min, allowing EVs to interact with the ligands on the biochip. After 8 min of reaction, 300 µL of regeneration buffer was injected at 200 µL/min for 1 min, followed by washing the biochip with running buffer. Most parameters were set automatically, with only the flow rate adjusted manually. The optimal flow rate for binding was 20-30 µL/min^[13]^. In this study, the working angle to produce total internal reflection was automatically set between 58.5° and 59°.

### SPRi data analysis

We used Scrubber software (BioLogic Software, Canberra, Australia) to analyze the SPRi data. The anti-mouse IgG antibody spot served as a negative control, so the signal intensities for analysis were calculated by subtracting the average signal from the anti-mouse IgG spot. Each ligand’s signal intensity was then normalized to the intensity of the anti-CD63 spot after subtracting the signal intensity of the isotype control.

### Preparing biochip for SPRi

The biochip included a glass prism and a thin gold layer modified with the activated carboxylic acid, which reacts with free amine groups on the ligand (SPRi-Biochip CS-HD, Horiba Scientific, Kyoto, Japan)^[13]^. We selected various antibodies and lectins for detecting specific EVs, as shown in Table 2. MAM lectin identifies structures with terminal sialic acid in α2,3-linkage (MAM lectin binding molecules: MBM)^[35]^. Mouse IgG isotype control was used as the negative control.

**Table 2:** Antibody and lectin information

**Target molecule**	**Company**	**Source**	**Clone**
CD63	FUJIFILM Wako	Mouse	3-13
CD9	FUJIFILM Wako	Mouse	1K
CD81	FUJIFILM Wako	Mouse	17B1
CD4	Becton Dickinson	Mouse	RPA-T4
CD8	Becton Dickinson	Mouse	RPA-T8
CD25	BioLegend	Mouse	BC96
CD20	BioLegend	Mouse	2H7
CD71	BioLegend	Mouse	CY1G4
CD44	BioLegend	Mouse	C44Mab-5
CD110	BioLegend	Mouse	S16017E
CD169	BioLegend	Mouse	7-239
EpoR	R&D Systems	Mouse	38407
AcvRIIA	R&D Systems	Mouse	126706
TfR2	R&D Systems	Mouse	353816
DARC	R&D Systems	Mouse	358307
Isotype control	Becton Dickinson	Mouse	MOPC-21
MBM	J-Chemical	-	-

EpoR: Erythropoietin receptor; AcvRIIa: activin receptor type-2A; TfR2: transferrin receptor 2; DARC: Duffy antigen receptor for chemokine; MBM: MAM lectin binding molecules.

Duplicate spots were prepared for each ligand, and after ligands binding, the biochip was incubated overnight with a 1% casein-PBS solution for blocking.

### Validation of EV purification

To validate EV purification, we used ExoQuick® as described and compared it with the conventional ultracentrifugation method^[36]^. Ultracentrifugation was performed at 120,000 × *g* for 80 min using Optima TLX (Beckman Coulter, CA, USA) and an Open-Top Thinwall Polypropylene Tube (Beckman Coulter). Transmission electron microscopy (TEM) analysis sample preparation and observation were conducted at Filgen, Inc. (Nagoya, Japan). Moreover, we quantified protein levels using a bicinchoninic acid assay (Nacalai Tesque) and performed western blotting for EV validation. The same EVs sample (15 µL of EVs solution lysed with RIPA buffer: 10 µg protein/lane) was electrophoresed on a 10% sodium dodecyl sulfate polyacrylamide gel under non-reducing conditions, and the separated proteins were transferred to a polyvinylidene difluoride (PVDF) membrane (Atto Co., Tokyo, Japan). The membranes were blocked for 60 min in EzBlock Chemi (Atto Co.). Primary antibodies for CD63 (1:1000; MBL, Tokyo, Japan) and CD81 (1:1000; MBL) were mixed and diluted in the blocking buffer and incubated overnight at 4 °C with the PVDF membrane. After washing the membrane three times with tris buffered saline with tween 20, it was incubated with a secondary horseradish peroxidase-conjugated anti-rabbit IgG antibody (1:1000; Abcam, Cambridge, UK) for 2 h at room temperature (almost 25 °C). The bound secondary antibodies were then detected using EzWestLumi Plus (Atto Co.) and a MultiImager II ChemiBOX (BioTools, Gunma, Japan).

### Statistical analysis

Uniform manifold approximation and projection (UMAP) and t-Distributed Stochastic Neighbor Embedding (t-SNE) were conducted using Python, and visualizations were created with Matplotlib. For UMAP, the parameters were set to 15 neighbors (the default value) and a minimum distance of 0.1 to achieve the clearest results. For t-SNE, the perplexity was set to 50. Comparisons between two groups (healthy controls *vs*. T2DM or HT) were made using the Student’s *t*-test, with *P* < 0.05 considered statistically significant. Pearson’s coefficients were used to evaluate correlations between molecule intensities. Multiple regression analysis was performed with IBM SPSS v.29 to obtain multiple correlation (R), R-squared, adjusted R-squared, and significance F for determining correlations among the three groups. Logistic regression analysis was also conducted using the same software. The sample size consisted of the number of samples treated during the study, with no a priori statistical sample size calculations performed. In this cross-sectional study, the sample size provided post hoc power calculations for each signal intensity between the two groups, with a two-sided significance level (α) of 0.05 [Supplementary Table 1]. Post hoc power tests were conducted using G^*^Power software (Heinrich-Heine-Universität Düsseldorf, Düsseldorf, Germany). All signal intensities are shown in Figure, but spots with insufficient post hoc power were excluded from statistical analysis.

## RESULTS

### Purification and detection of serum EVs by the SPRi method

**Figure 2:**
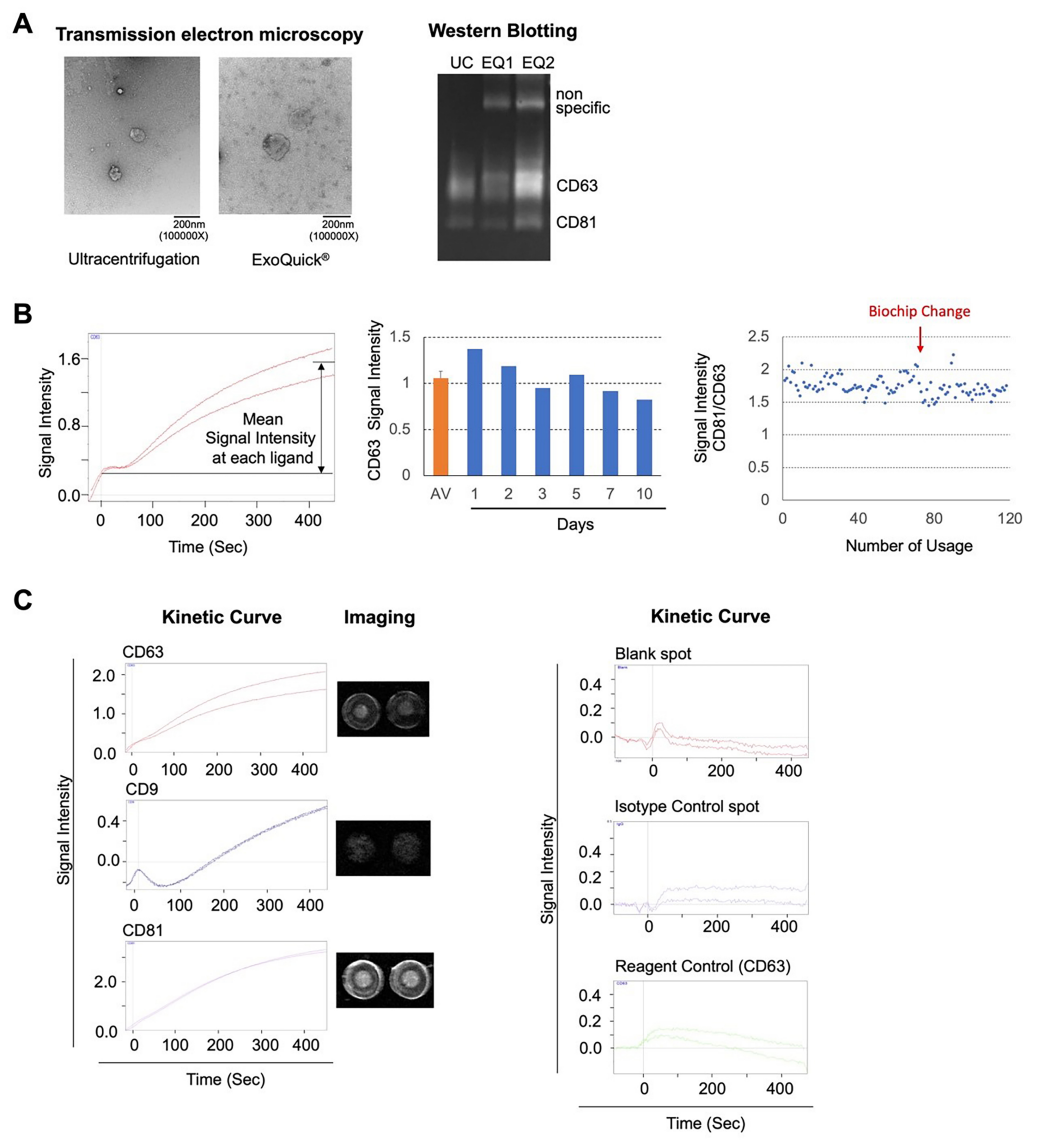
EV purification and detection using SPRi. (A) EVs were purified using ExoQuick®, and the extent of purification was assessed by TEM and western blotting, compared to ultracentrifugation; (B) Two spots were prepared for each ligand, and the average of the maximum values from the kinetic curves was used as the quantitative measure of the ligand; (C) Kinetic curve for tetraspanin family proteins (CD63, CD9, and CD81), blank and isotype control, and images of each spot obtained from measuring healthy human serum with SPRi. Reagent control (100-fold diluted ExoQuick) was used to confirm nonspecific reactions of the reagents. Evs: Extracellular vesicles; TEM: transmission electron microscopy; SPRi: surface plasmon resonance imaging.

EVs were purified from serum samples of healthy controls and patients with T2DM and HT using ExoQuick®, a method based on polyethylene glycol (PEG) precipitation. The purified EVs solution was then injected into the SPRi device flow system. The size of EVs and any coprecipitated debris were confirmed via TEM. In Western blotting, the ExoQuick samples were analyzed in duplicate, but the phoretic images were discordant, indicating contaminant protein and insufficient diffusion of EVs during resuspension and lysis. This precipitation method was employed in subsequent experiments because it was considered an appropriate technique for clinical testing. It effectively purified the same quantity of EVs more rapidly, though it produced more debris compared to ultracentrifugation [Figure 2A]. The time courses of EV binding to spotted anti-CD63 antibodies on the SPRi biochip are shown in Figure 2B (Left). Because SPRi signal intensities vary among different antigen-antibody complexes, a baseline was established at 0 min when the EVs solution was injected. The average intensity of each ligand spot at 8 min was recorded as the result of analyzing each EV surface antigen [Figure 2B]. Moreover, Figure 2B (Central) shows the signal intensity of CD63 when the same healthy control serum was measured repeatedly on the same biochip for 10 days. The signal intensity from day 2 to day 7 remained stable (mean: 1.02, upper limit: +0.088, lower limit: -0.098), although the signal intensity on day 1 was slightly elevated, likely due to the tendency for the initial measurement to yield higher values. The signal intensity remained reproducible for approximately 7 days but began to decrease gradually afterward. Figure 2B (Right) demonstrates that washing the biochip does not affect the reproducibility of signal intensity. However, the average signal intensity varied from one biochip lot to another. Based on these data, we utilized the same biochip for a maximum of one week or for up to 100 samples. Figure 2C displays the difference in maximum signal intensity at the anti-tetraspanin antibody spots. Signal intensities varied based on the antigen expression level on the EV surface and the reactivity with the antibody clone, with maximum signal intensities of 1.89 (CD63), 0.53 (CD9), and 3.31 (CD81) using healthy control serum. In addition, the signal intensities of the blank and isotype control spots are presented, along with the CD63 spot, which is measured using only the 100-fold dilution of the precipitation reagent. A signal intensity of approximately 0.1 was observed due to nonspecific reactions.

### Analysis of circulating EVs in patients with T2DM and HT using SPRi

**Figure 3:**
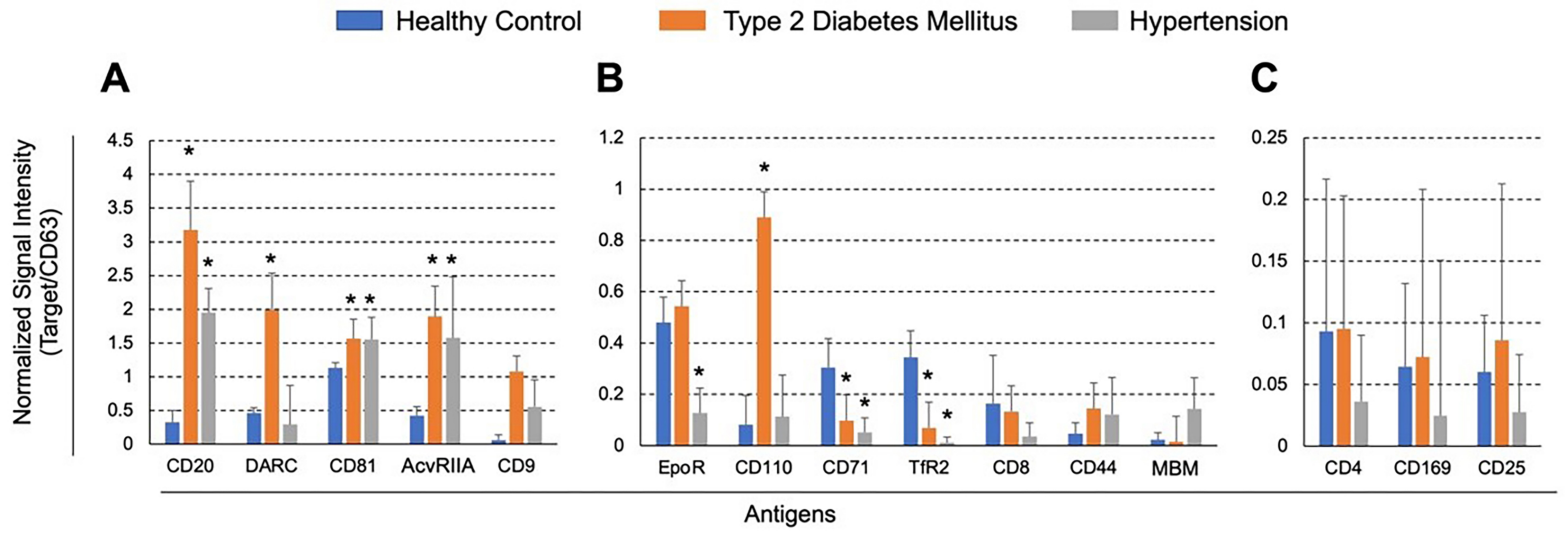
^*^Significant differences from control subjects (healthy controls) (*P* < 0.05). Each ligand’s measured value is normalized to the value of CD63. The columns represent healthy controls (blue), patients with T2DM (orange), and patients with HT (gray). (A) Antigens with high expression on the surface of any of the EVs. Antigens with (B) middle and (C) low expression on the surface of EVs. Values are shown as the mean ± SD. T2DM: Type II diabetes mellitus; HT: hypertension; Evs: extracellular vesicles.

Signal intensities from SPRi analysis were normalized against CD63, a common EV marker, to account for variations in EV recovery rates. The ratios of normalized intensities for each surface antigen revealed disease-specific profiles of circulating EVs in patients with T2DM and HT compared to normal subjects [Figure 3]. EVs with CD9 (+), CD81 (+), activin receptor type-2A (AcvRIIA) (+), CD20 (+), and CD44 (+) markers were elevated in patients with T2DM and HT [Figure 3A]. Duffy antigen receptor for chemokine (DARC) (+) and CD110 (+) EVs increased only in T2DM, whereas MBM (+) EVs were elevated only in HT. TfR2 (+) and CD71 (+) EVs decreased in both T2DM and HT, whereas EpoR (+) EVs decreased only in HT. On the other hand, the average signal intensities of CD4, CD169, and CD25 were below 0.1 in all groups [Figure 3C].

Disease-specific EV profiles were clearly demonstrated by comparing the relative increases in each marker-positive EVs in T2DM or HT to those in normal controls. In T2DM, EVs with CD9 (+), CD110 (+), CD20 (+), AcvRIIA (+), DARC (+), and CD44 (+) were elevated more than two-fold, and in HT, CD9 (+), MBM (+), CD20 (+), AcvRIIA (+), and CD44 (+) EVs showed similar increases [Table 3]. Four markers, CD9, CD20, AcvRIIA, and CD44, were upregulated in both T2DM and HT, with CD110 specifically elevated in T2DM and MBM in HT. This suggests that each disease has a distinct profile of circulating EVs.

**Table 3:** Profiles of EV markers in patients with T2DM and HT

**Healthy control**		**T2DM**		**HT**	
EV markers	Ratios^*^	EV markers	Ratios^*^	EV markers	Ratios^*^
CD81	1.131	CD20	3.178	CD20	1.950
EpoR	0.480	DARC	1.983	AcvRIIA	1.575
DARC	0.463	AcvRIIA	1.894	CD81	1.554
AcvRIIA	0.423	CD81	1.563	CD9	0.556
TfR2	0.344	CD9	1.078	DARC	0.292
CD20	0.326	CD110	0.890	MBM	0.143
CD71	0.305	EpoR	0.543	EpoR	0.127
CD8^**^	0.165	CD44	0.144	CD44	0.122
CD4^**^	0.093	CD8^**^	0.133	CD110	0.113
CD110	0.082	CD4^**^	0.111	CD71	0.052

^*^Ratios were calculated using normalized values with the intensity of anti-CD63 spots; ^**^Insufficient number of specimens determined by post hoc power analysis. EV: Extracellular vesicle; T2DM: type II diabetes mellitus; HT: hypertension; EpoR: erythropoietin receptor; DARC: Duffy antigen receptor for chemokine; AcvRIIa: activin receptor type-2A; TfR2: transferrin receptor 2; MBM: MAM lectin binding molecules.

### Analysis of SPRi data using dimensionality reduction techniques

**Figure 4:**
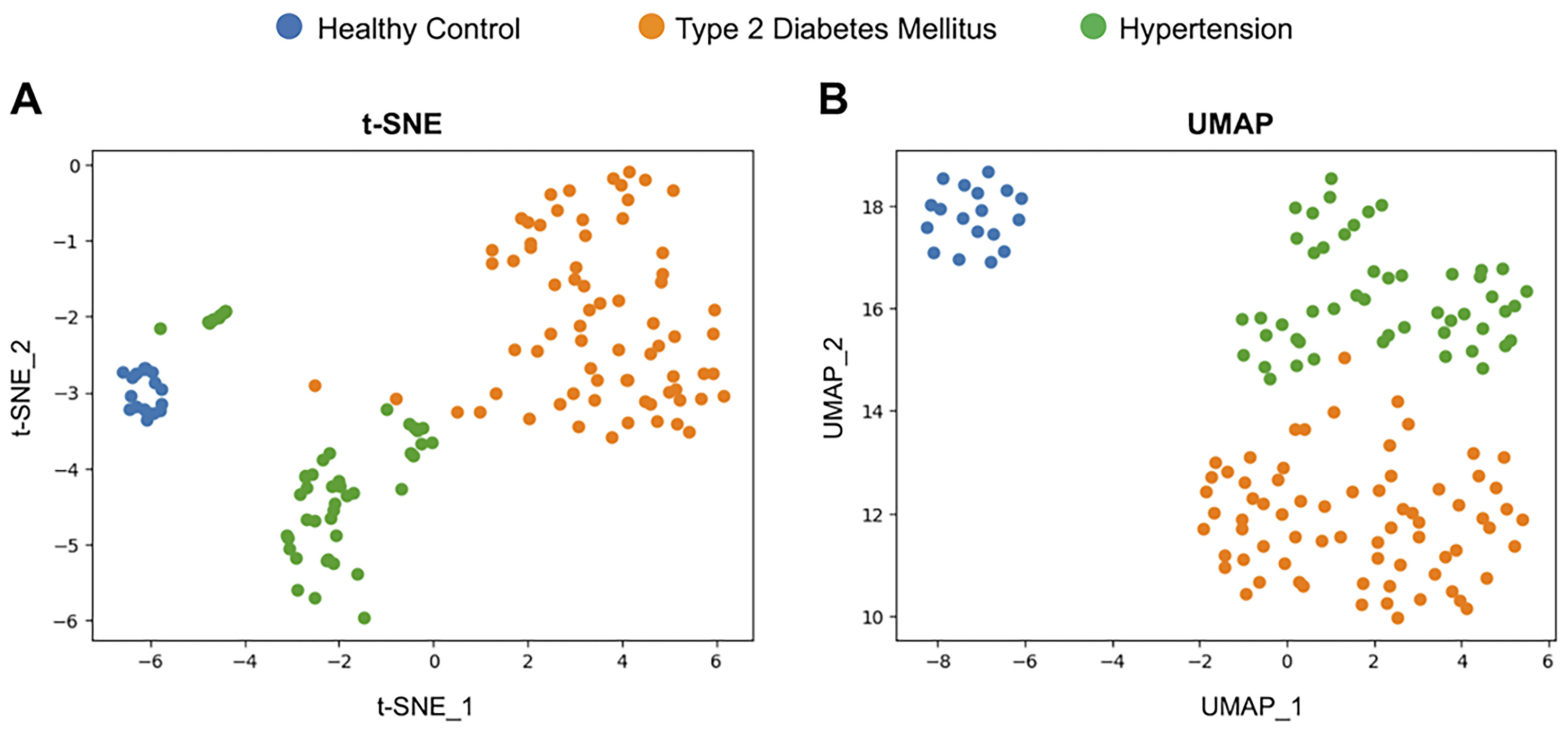
Cluster formation of each disease group using dimensionality reduction techniques. Visualization is provided by two dimensionality reduction techniques. Each node represents healthy controls (blue), patients with T2DM (orange), and patients with HT (green). (A) t-SNE method and (B) UMAP method. T2DM: Type II diabetes mellitus; HT: hypertension; t-SNE: t-Distributed Stochastic Neighbor Embedding; UMAP: uniform manifold approximation and projection.

We used t-SNE and UMAP analyses to differentiate the profiles of circulating EVs among patients with HT or T2DM and control subjects. Under optimal conditions, T2DM, HT, and control subjects formed distinct clusters [Figure 4A]. UMAP analysis produced more integrated plots compared to t-SNE [Figure 4B]. These results indicate that patients with T2DM or HT have distinct circulating EV profiles and demonstrate that SPRi is an effective technology for disease identification.

### A set of three EV antigens can distinguish between T2DM, HT, and control subjects

**Figure 5:**
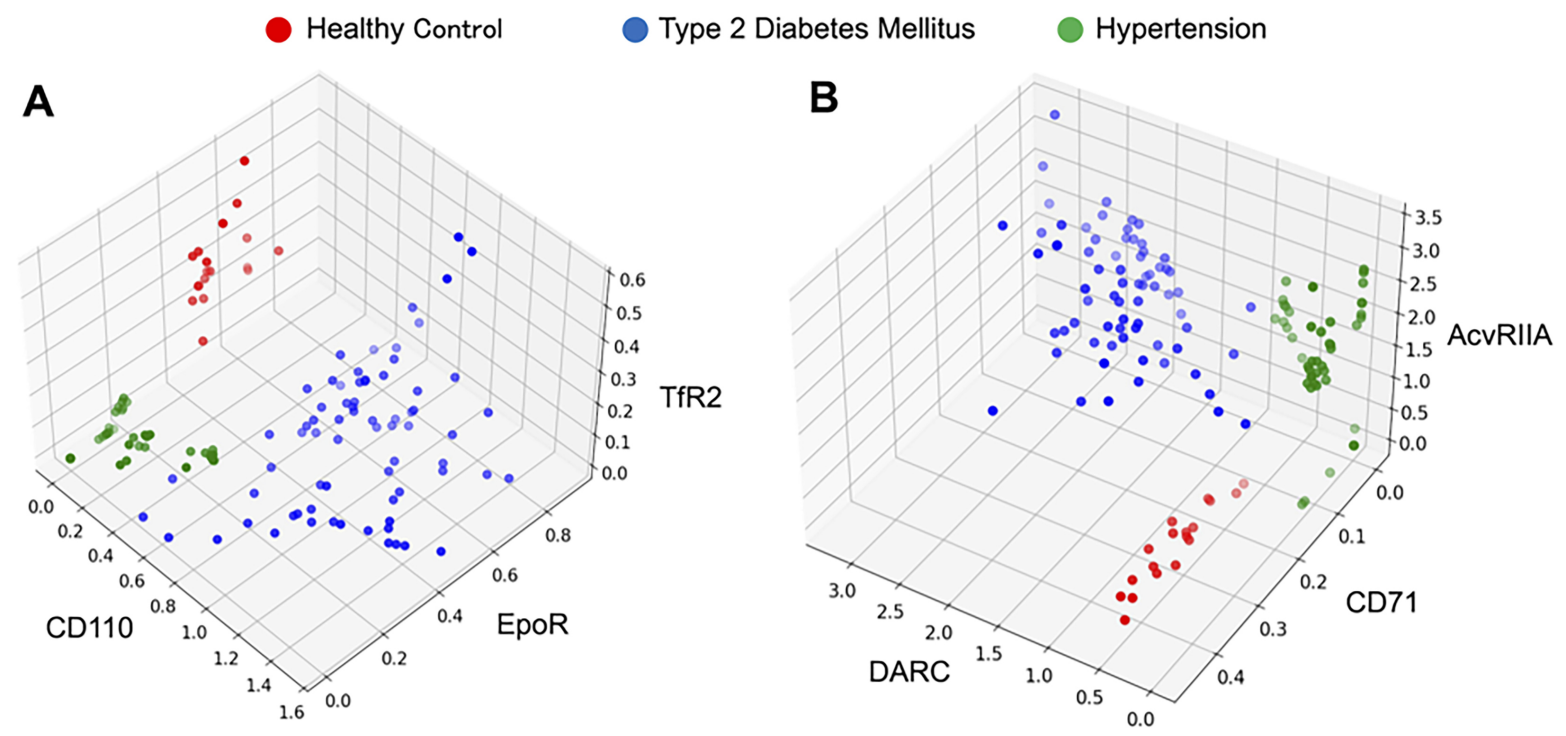
Antigens with distinct variations can differentiate between disease groups. The 3D plot displays the normalized signals of various antigens measured by SPRi. Node represents healthy controls (red), patients with T2DM (blue), and patients with HT (green). (A) CD110, TfR2, and EpoR; (B) DARC, AcvRIIA, and CD71. SPRi: Surface plasmon resonance imaging; T2DM: type II diabetes mellitus; HT: hypertension; DARC: Duffy antigen receptor for chemokine; AcvRIIA: activin receptor type-2A.

Based on these results of the profiles of circulating EVs specific to each disease, to create a clinically useful SPRi-based EVs assay, we evaluated 12 different sets of three EV markers for their ability to differentiate T2DM, HT, and control subjects. After preliminary testing all sets, we selected two effective sets with the ability to separate three groups. The combination of CD110, EpoR, and TfR2 clearly distinguished T2DM, HT, and control groups [Figure 5A], as did DARC, CD71, and AcvRIIA [Figure 5B]. Other marker sets did not provide clear separation (data not shown). These findings indicate high expression of CD110 and DARC on EVs in T2DM and low expression of EpoR on EVs in both diseases [Figure 3 and Table 2]. Thus, using these sets of three antigens for SPRi analysis may be useful for diagnosing T2DM and HT.

### Correlation with the disease severity

**Figure 6:**
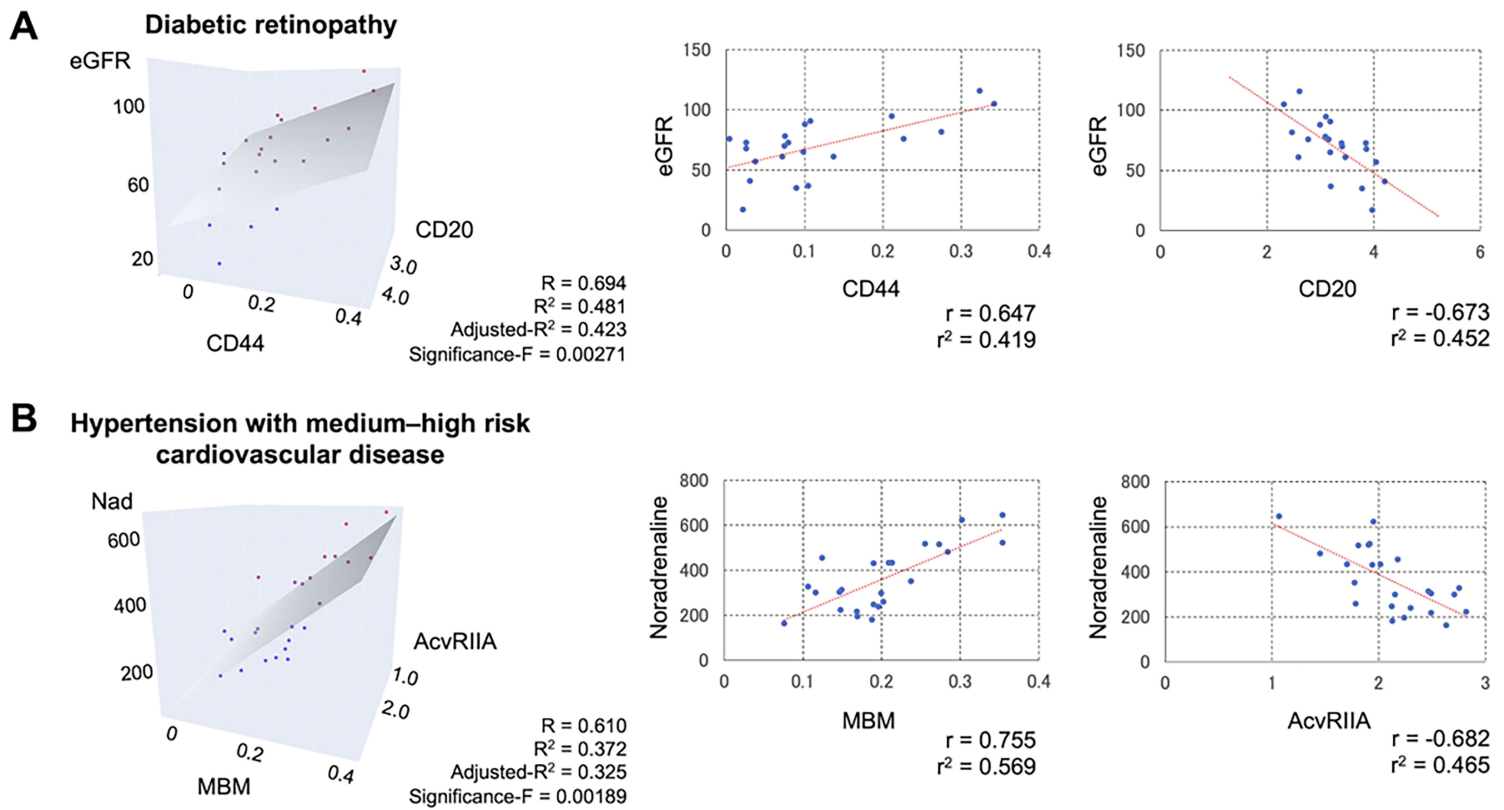
Signal intensity changes of various antigens on the EV surface partially correlate with disease severity. (A) Correlation between eGFR values and signal intensities of CD20 and CD44 on the EV surface in patients with diabetic retinopathy; (B) Correlation between noradrenaline levels and signal intensities of AcvIIRA and MBM on the EV surface in patients with moderate- or high-risk cardiovascular disease. R and R^2^ represent the correlation coefficient and R-squared, respectively. EV: Extracellular vesicle; eGFR: estimated glomerular filtration rate; AcvRIIA: activin receptor type-2A; MBM: MAM lectin binding molecules.

We examined whether the profile of circulating EVs correlated with the clinical data for each disease. In T2DM patients with retinopathy, the estimated glomerular filtration rate (eGFR) showed a significant correlation with CD44 (+) and CD20 (+) EVs [Figure 6A]. However, no correlation was noted between EV intensities and diabetic nephropathy [Supplementary Table 2]. In patients with moderate- or high-risk HT, serum noradrenaline levels were positively correlated with MBM (+) EVs and negatively correlated with AcvRIIA (+) EVs [Figure 6B]. Logistic regression analysis also indicated an association between these EV antigens and the disease [Supplementary Table 3]. Multiple regression analysis, including confounding factors such as age and gender, showed that these EV antigens were correlated with eGFR and noradrenaline [Table 4].

**Table 4:** Profiles of EV markers in patients with T2DM and HT

	**T2DM with retinopathy (*n* = 21)**					
	Beta	*t*	*P*	95%CI	
**eGFR**					
(Constant)		3.68	0.002	61.04	227.44
Ages	-0.27	-2.21	0.042^*^	-1.39	-0.03
Gender	0.19	1.55	0.141	-3.73	23.98
CD20	-0.43	-2.19	0.043^*^	-34.82	-0.59
CD44	0.49	2.53	0.022^*^	18.10	204.32
AcvRIIa	-0.04	-0.34	0.741	-15.72	11.42
MBM	0.15	1.19	0.253	-104.4	369.8
	**T2DM with Nephropathy (*n* = 20)**					
	Beta	*t*	*P*	95%CI	
**eGFR**					
(Constant)		3.69	< 0.001	59.42	202.37
Ages	-0.52	-3.98	< 0.001^*^	-1.56	-0.51
Gender	0.29	2.25	0.030^*^	1.68	30.71
CD20	-0.04	-0.20	0.840	-15.34	12.57
CD44	-0.01	-0.05	0.964	-79.61	76.11
AcvRIIa	-0.06	-0.45	0.656	-20.91	13.30
MBM	0.01	0.03	0.975	-255.73	263.77
	**HT - moderate/high-risk group (*n* = 22)**					
	Beta	*t*	*P*	95%CI	
**Noradrenaline**					
(Constant)		1.77	0.096	-84.82	948.14
Ages	0.16	1.40	0.178	-0.91	4.55
Gender	-0.09	-0.75	0.462	-94.18	44.56
CD20	-0.01	-0.10	0.920	-116.33	105.63
CD44	-0.09	-0.79	0.441	-1,017.33	462.27
AcvRIIa	-0.41	-2.26	0.036^*^	-220.04	-8.12
MBM	0.54	3.27	0.004^*^	33.43	153.41
	**HT - low-risk group (*n* = 25)**					
	Beta	*t*	*P*	95%CI	
**Noradrenaline**					
(Constant)		4.66	< 0.001	230.45	618.48
Ages	-0.02	-0.08	0.937	-1.62	1.50
Gender	-0.48	-2.15	0.049^*^	-93.87	-0.29
CD20	-0.32	-1.33	0.204	-93.98	21.827
CD44	0.14	0.49	0.628	-180.30	289.18
AcvRIIa	-0.52	-2.19	0.044^*^	-93.28	-1.42
MBM	-0.21	-0.85	0.410	-371.10	160.02

^*^P < 0.05. Beta: standardized regression coefficient; *t*: *t*-statistic; EV: extracellular vesicle; T2DM: type II diabetes mellitus; HT: hypertension; CI: confidence interval; eGFR: estimated glomerular filtration rate; AcvRIIa: activin receptor type-2A; MBM: MAM lectin binding molecules.

## DISCUSSION

Recent research has highlighted the clinical significance of circulating EVs in various diseases^[37,38]^. In this study, we used the SPRi method to analyze circulating blood cell-derived EVs in conditions associated with microvascular damages as common pathophysiologic complications. Tetraspanins, which are commonly found on the surface of EVs, interact with various transmembrane and cytoplasmic signaling proteins^[39-42]^, making them key markers for identifying EVs through anti-CD63, CD9, and CD81 antibodies^[2,4]^. Therefore, we normalized EV profiles based on the number of CD63 (+) EVs. Our findings revealed that T2DM and HT have distinct EV profiles; T2DM showed a mixed pattern of commonly upregulated EVs [CD9 (+), CD20 (+), AcvRIIA (+), CD44 (+)] along with disease-specific upregulated EVs, such as CD110 (+), whereas HT displayed MBM (+) EVs.

CD9 has various biological roles depending on its binding partners, leading to variable expression levels across different pathological conditions, including cellular adhesion, migration, tumorigenesis, inflammation, and cellular senescence^[43-45]^. Given that T2DM and HT are risk factors for atherosclerosis, our data may reflect damage to vascular endothelial cells caused by atherosclerosis in these diseases. Further research with large cohorts and comprehensive clinical data is needed. Our results showed a notable increase in CD20 (+) EVs in both T2DM and HT. Macroangiopathy is a major cause of mortality in T2DM^[46,47]^, and the presence of CD20 (+) lymphocytes and CD68 (+) macrophages is associated with neovascularization of the femoral artery in patients with T2DM^[47]^. B cells play a role in the development of type 1 diabetes mellitus and diabetic kidney disease^[48]^. In patients with T2DM, elevated serum IL-6 levels, which stimulate B cell functions, were associated with kidney damage^[49]^. Focusing on inflammation, we categorized the patients’ C-reactive protein (CRP) levels based on the presence or absence of complications. The median CRP values for retinopathy, nephropathy, and no complications were 0.27, 0.21, and 0.09, respectively. In addition, the correlation coefficients between CRP and CD20 signal intensity for these groups were 0.57, 0.59, and 0.59, respectively (Dara not shown). Although further investigation is necessary, as CRP responds to systemic inflammation and many patients with T2DM have urinary tract infections or other inflammatory conditions, the increase in CD20 (+) EVs observed in these patients may indicate an inflammatory process related to atherosclerosis. Furthermore, our data revealed higher expression of AcvRIIA on EVs in patients with T2DM and HT. AcvRIIA binds activin A, which is elevated in diseases associated with microangiopathy, including T2DM^[50]^, T2DM with coronary heart disease^[51]^, metabolic syndrome with HT^[52]^, and heart failure^[53]^. Activin A promotes AcvRIIA expression^[54]^ and erythroid differentiation through AcvRIIA^[55]^. According to The Human Protein Atlas (https://www.proteinatlas.org), AcvRIIA is highly expressed in skin cells but is not specific to any particular tissue. Although the specific cells and tissues responsible for the increase in AcvRIIA (+) EVs are not identified, our study suggests that these EVs could serve as potential biomarkers for microangiopathy complicated with T2DM and HT. CD110 (+) EVs were elevated only in T2DM in our study. CD110 is a thrombopoietin receptor found on megakaryocytes and platelets. Thrombopoietin binds to CD110, promoting the production of immature and activated platelets in blood^[56,57]^. Activated platelets release EVs that can damage vascular endothelial cells^[58-60]^. Previous research by Larsen *et al*. found significantly increased thrombopoietin levels in diabetic patients^[61]^, which aligns with our finding of a significant increase in CD110 (+) EVs in T2DM [Figure 3]. Thus, the SPRi method may be useful for evaluating platelet-related risks in T2DM. Moreover, the miRNA profile in platelet-derived EVs has been proposed as a relevant marker for predicting T2DM complications^[62]^. Further research into the contents of these EVs is recommended to better understand the pathogenetic roles of CD110 (+) EVs in T2DM.

CD44 (+) EVs were more than twice as abundant in T2DM and HT [Table 2] and were positively correlated with eGFR levels in T2DM patients with retinopathy [Figure 6A]. CD44 is a surface glycoprotein found on macrophages and T cells^[63]^. Kodama *et al.* found a positive relationship between soluble CD44 protein and HbA1c levels and insulin resistance in T2DM, noting its association with adipose tissue inflammation^[64]^. CD44 aids leukocyte adhesion to endothelial cells and stimulates chemokine release from macrophages, which promotes the proliferation and migration of vascular smooth muscle cells^[65-67]^. It is not clear if soluble CD44 proteins are cargo of EVs. Although CD44 (+) EVs may influence vascular function, the purification method used in this study did not completely remove non-EVs CD44 protein. As SPRi analysis may detect non-EVs CD44 protein, using a more refined purification method is recommended to confirm these findings. In addition, CD44 and CD20 did not correlate with biochemical data in patients with diabetic nephropathy. Lu *et al*. suggested that urinary EVs may be more effective for diagnosing diabetic nephropathy^[68]^, as urine could be a better medium than serum for studying kidney disease due to the presence of urinary toxins and electrolyte abnormalities.

Our study is the first to reveal changes in EVs with antigens associated with erythroid lineage cells in T2DM. Originally identified as a blood group antigen on erythrocytes^[69]^, DARC is classified as an atypical chemokine receptor 1 (ACKR1), a member of the ACKR family. This transmembrane protein does not interact with signal transduction pathways but functions through the uptake of extracellular molecules into the cytoplasm, scavenging, and transferring ligands^[70,71]^. Recent research also shows that DARC is expressed on vascular endothelium and acts as a chemokine receptor^[72]^. In ApoE-knockout mice on a high-fat diet, ACKR1 was overexpressed on red blood cells (RBCs), which accelerated atherosclerotic changes^[70]^. Our findings suggest that DARC (+) EVs could serve as useful biomarkers for detecting vascular endothelial damage in T2DM.

Our results showed a significant reduction in CD71 (+) and TfR2 (+) EVs in patients with T2DM. CD71 is equivalent to TfR1, and both TfR1 and TfR2 are transferrin-binding proteins^[60]^. TfR1 is generally found on cell surfaces and facilitates the uptake of transferrin-bound iron into cells, whereas TfR2 is specifically expressed on early erythroid progenitors, promoting RBC production through a complex with EpoR^[73]^. Our study identified CD20 (+), DARC (+), AcvRIIA (+), and CD110 (+) as the main populations of circulating EVs in T2DM, suggesting that most circulating EVs are non-erythroid. This may explain the relative decrease in CD71(+) and TfR2 (+) EVs. These findings highlight the need for precise nanoparticle counting to accurately determine EV numbers in each sample. Combining EV counting with SPRi technology is essential for a thorough understanding of the mechanisms behind the decrease in CD71 (+) and TfR2 (+) EVs.

SPRi analysis offers three key advantages: First, it requires only a small volume (150 µL) of serum. Second, costs are lower because the same biochip can be reused up to almost 100 times after stripping EVs bound to the immobilized antibody. Third, it allows for simultaneous analysis of 48 different EV markers, even if each marker is tested in duplicates^[13]^. The sensitivity of SPRi is comparable to ELISA and can be enhanced by using a two-antibody sandwich assay^[19,74]^. Although ELISA is a widely used and effective technique in clinical laboratories, it is costly due to its single-use nature. In contrast, SPRi allows for repeated measurements through biochip regeneration, making it more cost-effective and capable of comprehensive quantitative analysis of up to 96 antigens. Briefly, the cost is approximately $5 per sample for the SPRi in this study, approximately $7 per sample per antigen for the research ELISA, and $10~ per sample for the clinical ELISA. In terms of clinical applications, integrating SPRi with specialized analysis software is highly effective. This combination enables a variety of operations, including disease classification through dimensionality reduction and severity assessment using specific antigens in a single specimen measurement. In this study, 25 EV antigens clearly separated T2DM, HT, and control subjects [Figure 4]. The time required to measure a single specimen is approximately 15 min, making it suitable for clinical testing. SPRi is also useful as a discovery tool to search for disease-related antigens. Two sets of EV markers [(CD110, EpoR, and TfR2) and (DARC, CD71, and AcvRIIA)] were effective in distinguishing control subjects from those with T2DM and HT [Figure 5]. As all these antigens are expressed by hematopoietic cells, it suggests that blood cell-derived EVs vary among the diseases studied. Further efforts are needed to identify effective EV markers for more accurate and sensitive analysis. Preventing serious cardiovascular disease is a crucial clinical strategy. Our study found that AcvRIIA (+) EVs had a significant negative correlation with serum noradrenaline levels, which are associated with atherogenesis^[75]^. However, MBM (+) EVs showed a significant positive correlation with noradrenaline levels. In patients with HT, the high sialic acid content in glycolipids from erythrocyte membranes may explain the increased MBM expression^[76]^, as it is likely due to erythrocyte-derived EVs with elevated sialic acid content. Compared to other conventional protein analysis methods such as western blotting, ELISA, and flow cytometry, the primary advantage of SPRi is its label-free detection. This feature eliminates the need to account for antibody labeling (fluorescence, enzyme) and its combination for protein detection. Of course, nano-flow cytometry has the advantage of measuring both particle number and size. Although quantitative PCR (qPCR) is an important method for utilizing EVs as biomarkers because of its high detection sensitivity, quantitative internal controls have not yet been established, and multiple non-coding RNAs need to be measured for effective biomarker application. SPRi does not require the extraction of nucleic acids and can analyze multiple specific antigens in a single measurement.

The primary limitation of this study is the small sample size, particularly the number of healthy controls. In Japan, HT rates exceed 30% in people in their 40s, 50% in their 50s, and 60% in their 60s^[77]^. In addition, finding truly healthy controls of various ages who do not have T2DM or other diseases proved challenging. Although post hoc power analysis confirmed the power of each spot compared to the control, larger cohorts are needed to validate these results. Another issue is the EV purification method. The PEG-based precipitation used in this study also precipitates proteins and lipids, potentially increasing debris in patients with hyperlipidemia and hyperglobulinemia, which can affect ligand reactivity and cause nonspecific responses. In fact, as shown in Figure 2A, lipoproteins and contaminant proteins were not removed even though large vesicles were removed by the 0.22 µm filter. This resulted in some nonspecific reactions, as shown in Figure 2C. A similar effect has been observed at this point by Hosseinkhani *et al.* ^[78]^. This may result in decreased sensitivity and an inability to detect low amounts of specific EVs. However, as reported by Hirschberg *et al*., the isolation of EVs using size-exclusion chromatography (SEC) results in high purity but requires ultrafiltration for SPRi, which raises the cost of clinical testing^[79]^. Improved low-cost methods for high-yield and high-purity EV purification are necessary. Additionally, we confirmed EVs by CD63/CD81 protein and TEM according to MISEV guidelines^[80]^, and our TEM data indicated that the sizes of the sample EVs were relatively homogeneous in this study. However, EVs are heterogeneous vesicle groups, such as exosomes, ectosomes, microvesicles, and Exosome-like vesicles^[80]^; there was an overlap in particle sizes among the EVs groups. Although we isolated EVs by precipitation method following filtration through a 0.22 µm filter, there may be heterogeneity of size of EVs between 200 and 220 nm because the definition of small EVs is < 200 nm. Our data require validation to confirm the purity of EVs through particle size analysis in further study to perform more precise quantification. It was also the limitation of this study that we could not confirm the quality of EV preparations using CD9 protein and calnexin, which are the markers to estimate EV purity^[81]^. In addition, the distribution of amino groups, particularly lysine residues, throughout the antibody raises concerns about the uniformity of antibody binding to the biochip. It is possible to address this problem by coating biochips and modifying antibodies, which presents a significant challenge for our future endeavors. As other potential external factors, the dysregulated release of EVs from damaged organs, as a complication of the diseases, influences EV signatures. Recently, bacterial EVs in circulating blood have been highlighted as a biomarker for infectious diseases^[82]^, whereas these vesicles can affect the signature of human EVs through nonspecific reactions. This study focused on T2DM and HT, which are major diseases that promote atherosclerosis. Further EVs analysis in various diseases is needed to determine diseases-specific EV signatures by the SPRi technology.

In conclusion, we used SPRi to analyze serum EVs from patients at high risk for atherosclerosis and demonstrated the clinical value of the EV signatures obtained. Our findings indicated that the EV signatures vary between T2DM and HT and correlate with disease progression. Notably, changes in blood cell-derived EVs were prominent in these patients. Although blood cell-derived EVs can fluctuate in hematopoietic diseases^[83]^ and immune disorders^[84]^, our study showed that these EVs also change in patients with T2DM and HT independently of such conditions. Most current tests for T2DM assess tissue damage that has already occurred, and tests for HT are not adequately predictive of disease progression. If EVs released during cellular injury could be detected before tissue damage, they could serve as biomarkers for the early detection of disease progression and the prediction of tissue damage. That is, the use of EV as a biomarker has the potential to improve prognostic factors compared to current clinical tests. If adopted clinically, the SPRi method could enable a comprehensive and cost-effective analysis of EV surface markers with high sensitivity. Our study highlights the potential of SPRi for profiling blood cell-derived circulating EVs as a new tool for diagnosing and monitoring various diseases.

## DECLARATIONS

### Availability of data and materials

The datasets generated and/or analyzed during the current study are available from the corresponding author upon reasonable request.

### Copyright

© The Author(s) 2025.
