# Supplementary material for: Disease-specific signatures of circulating extracellular vesicles detected by the surface plasmon resonance imaging: a pilot study
Source: Extracell Vesicles Circ Nucl Acids. 2025 Feb 11;6(1):36–53. doi: 10.20517/evcna.2024.82 (PMC11977349; doi:10.20517/evcna.2024.82)
Supplement: Supplementary file 1 [file evcna-6-1-36-SupplementaryMaterials.pdf]

**Supplementary Materials**

# Disease-specific signatures of circulating extracellular vesicles detected by the surface plasmon resonance imaging: a pilot study

**Tatsuki Shibuta[1], Yukichi Takada[1], Shiori Nishinosono[1], Seiko Yasuda[1], Yasuhiro Ono[2], Yoshitaka Hirooka[1,3,4], Daisuke Irikura[5], Kensuke Saito[5], Tsukuru Umemura[1,4]**

[1]Department of Medical Technology and Sciences, International University of Health and Welfare, Fukuoka 831-8501, Japan.
[2]Department of Diabetes and Metabolism, Kouhoukai Takagi Hospital, Fukuoka 831-0016, Japan.
[3]Hypertension and Heart Failure Center, Kouhoukai Takagi Hospital, Fukuoka 831-0016, Japan.
[4]Graduate School, International University of Health and Welfare, Fukuoka 831-8501, Japan.
[5]HORIBA, Ltd, Kyoto City, Kyoto 601-8510, Japan.

**Correspondence to:** Prof. Tatsuki Shibuta, Department of Medical Technology and Sciences, International University of Health and Welfare, 137-1 Enokizu, Okawa City, Fukuoka 831-8501, Japan. E-mail: tshibuta@iuhw.ac.jp; Dr. Tsukuru Umemura, Graduate School, International University of Health and Welfare, 137-1 Enokizu, Okawa City, Fukuoka 831-8501, Japan. E-mail: umemura@iuhw.ac.jp

**Supplementary Table 1. Post hoc power analysis**

| EV markers | T2DM | | | HT | |
|---|---|---|---|---|---|
| | Effect size | Power | n needed for 80% power | Effect size | Power |
| CD20 | 5.44 | 1.00 | - | 5.76 | 1.00 |
| DARC | 3.85 | 1.00 | - | 0.83 | 0.84 |
| CD81 | 2.02 | 1.00 | - | 2.06 | 1.00 |
| AcvRIIa | 4.43 | 1.00 | - | 5.61 | 1.00 |
| CD9 | 5.93 | 1.00 | - | 1.72 | 1.00 |
| EpoR | 0.60 | 0.62 | - | 3.60 | 1.00 |
| CD110 | 4.16 | 1.00 | - | 0.80 | 0.81 |
| CD71 | 2.07 | 1.00 | - | 2.87 | 1.00 |
| TfR2 | 2.59 | 1.00 | - | 3.75 | 1.00 |
| CD44 | 1.03 | 0.97 | - | 0.71 | 0.71 |
| MBM | 0.95 | 0.94 | - | 2.75 | 1.00 |
| CD4 | 0.16 | 0.09* | 635 | 0.81 | 0.82 |
| CD8 | 0.19 | 0.11* | 420 | 0.94 | 0.91 |
| CD25 | 0.27 | 0.17* | 217 | 0.70 | 0.70 |
| CD169 | 0.07 | 0.06* | 3102 | 0.78 | 0.79 |

*If power is less than 0.6, the required sample size is calculated. T2DM: Type II diabetes mellitus; HT: hypertension; DARC: Duffy antigen receptor for chemokine; AcvRIIa: activin receptor type-2A; EpoR: erythropoietin receptor; TfR2: transferrin receptor 2; MBM: MAM lectin binding molecules.

**Supplementary Table 2. Correlation analysis between EV markers and each biochemical test data**

| | **T2DM** | | | | | | | | | | | | **HT** | |
|---|---|---|---|---|---|---|---|---|---|---|---|---|---|---|
| | **Retinopathy** | | | | | | **Nephropathy** | | | | | | Moderate / High risk Group (n = 22) | Low risk Group (n = 25) |
| | Yes (n = 21) | | | No (n = 50) | | | Yes (n = 20) | | | No (n = 51) | | | | |
| markers | eGFR | sCre | U-Alb | eGFR | sCre | U-Alb | eGFR | sCre | U-Alb | eGFR | sCre | U-Alb | Noradrenaline | |
| CD20 | ***-0.673*** | 0.285 | 0.121 | 0.022 | 0.147 | 0.136 | 0.078 | 0.223 | 0.083 | -0.030 | 0.025 | -0.033 | 0.374 | -0.303 |
| DARC | -0.144 | -0.008 | -0.072 | -0.128 | 0.389 | 0.194 | -0.105 | 0.345 | 0.209 | -0.121 | 0.062 | -0.071 | 0.226 | 0.003 |
| CD81 | -0.310 | 0.073 | -0.046 | 0.037 | 0.205 | 0.193 | 0.070 | 0.201 | 0.099 | 0.030 | -0.079 | 0.100 | -0.173 | -0.304 |

| | | | | | | | | | | | | | | |
|---|---|---|---|---|---|---|---|---|---|---|---|---|---|---|
| AcvRIIa | 0.172 | -0.046 | -0.081 | -0.038 | 0.209 | 0.055 | 0.067 | 0.126 | -0.073 | 0.036 | 0.013 | -0.056 | ***-0.682*** | -0.149 |
| CD9 | 0.200 | -0.097 | -0.162 | -0.206 | 0.397 | 0.167 | -0.014 | 0.173 | -0.013 | -0.088 | 0.150 | -0.012 | -0.084 | -0.269 |
| EpoR | 0.085 | 0.072 | -0.037 | -0.106 | -0.060 | -0.271 | 0.104 | -0.003 | -0.342 | -0.263 | 0.328 | 0.002 | 0.056 | -0.225 |
| CD110 | 0.077 | -0.223 | -0.153 | -0.109 | 0.170 | 0.040 | -0.097 | 0.014 | -0.049 | -0.045 | -0.016 | 0.159 | 0.083 | -0.126 |
| CD71 | -0.103 | 0.043 | 0.213 | -0.029 | 0.062 | 0.009 | -0.176 | 0.010 | 0.030 | 0.146 | 0.001 | 0.056 | 0.102 | 0.042 |
| TfR2 | 0.213 | -0.003 | -0.154 | -0.202 | -0.017 | -0.148 | -0.039 | -0.038 | -0.255 | -0.118 | 0.239 | -0.134 | 0.018 | 0.062 |

| | | | | | | | | | | | | | | |
|---|---|---|---|---|---|---|---|---|---|---|---|---|---|---|
| CD44 | ***0.647*** | -0.133 | -0.131 | -0.081 | -0.126 | -0.175 | 0.049 | -0.243 | -0.279 | -0.012 | 0.154 | -0.051 | 0.050 | -0.152 |
| MBM | -0.078 | -0.247 | -0.109 | 0.082 | 0.151 | 0.293 | 0.167 | -0.086 | 0.184 | 0.030 | -0.177 | 0.007 | ***0.755*** | -0.126 |

The values in the table indicate correlation coefficients (r). Bold and italicized numbers are those with higher correlation coefficients than others. T2DM: Type II diabetes mellitus; HT: hypertension; eGFR: estimated Glomerular Filtration Rate; sCre: serum Creatinine; U-Alb: Urine-Albumin; DARC: Duffy antigen receptor for chemokine; AcvRIIa: activin receptor type-2A; EpoR: erythropoietin receptor; TfR2: transferrin receptor 2; MBM: MAM lectin binding molecules.

**Supplementary Table 3. Logistic regression analysis results on laboratory data and SPRi intensities in T2DM and HT patients**

| | **T2DM** | | | | **HT** | |
|---|---|---|---|---|---|---|
| | Retinopathy (n = 21) | | Nephropathy (n = 20) | | Moderate/High risk Group (n = 22) | |
| Variables | OR (95% CI) | *P* | OR (95% CI) | *P* | OR (95% CI) | *P* |
| Ages | 0.996 (0.94 - 1.05) | 0.873 | 1.022 (0.93 - 1.12) | 0.637 | 1.010 (0.95 - 1.08) | 0.763 |
| Gender | 0.516 (0.16 - 1.71) | 0.279 | 1.656 (0.38 - 2.10) | 0.101 | 2.179 (0.33 - 1.44) | 0.419 |
| eGFR | 0.991 (0.97 - 1.02) | 0.461 | 1.133 (1.03 - 1.32) | 0.088 | | |
| sCre | 1.263 (0.66 - 2.41) | 0.479 | 0.946 (0.47 - 1.63) | 0.097 | | |
| U-Alb | 1.001 (0.99 – 1.01) | 0.398 | 1.074 (1.02 - 1.13) | ***0.005**** | | |
| CD20 | 0.315 (0.09 - 1.09) | **0.067** | 1.169 (0.41 - 2.59) | 0.237 | | |
| CD44 | 0.002 (0.00 – 1.08) | **0.053** | 0.809 (0.25 - 0.87) | 0.414 | | |
| BNP | | | | | 0.997 (0.95 - 1.05) | 0.965 |
| Nad | | | | | 1.023 (1.01 - 1.04) | ***0.003**** |
| AcvRIIa | | | | | 0.070 (0.00 - 0.24) | **0.063** |
| MBM | | | | | 0.036 (0.00 - 0.91) | ***0.022**** |

*$P < 0.05$. Bold numbers without asterisks are low P-values that did not achieve the 0.05 criterion. T2DM: Type II diabetes mellitus; HT: hypertension; OR: odds ratio; CI: confidence interval; eGFR: estimated Glomerular Filtration Rate; sCre: serum Creatinine; U-Alb: Urine-Albumin; BNP: brain natriuretic peptide; Nad: noradrenaline; AcvRIIa: activin receptor type-2A; MBM: MAM lectin binding molecules.
